# HeartMath Coherence Model Throws New Light on Arka Dhyana Intuitive Meditation

**DOI:** 10.3390/healthcare9091162

**Published:** 2021-09-05

**Authors:** Tina Lindhard, Caroll Hermann, Stephen D. Edwards

**Affiliations:** 1 Department of Psychology, International University of Professional Studies, Mau, HI 96768, USA; 2 Department of Psychology, University of Zululand, Richards Bay 3886, South Africa; HermannC@unizulu.ac.za (C.H.); profsdedwards@gmail.com (S.D.E.)

**Keywords:** HeartMath, coherence, Intuitive Meditation, levels of consciousness, Inner Balance technology, heart centre, feeling, self, inner being, embryogenesis

## Abstract

In an experimental evaluation of an introductory Arka Dhyana (Intuitive Meditation) course, HeartMath (HM) Inner Balance or emWave2 electronic technology showed highly significant increases in both coherence and achievement in six participants who learned how to change their level of consciousness as proposed by the Theory of the Six Main Levels of Consciousness. During the course, which was offered to an international audience via Zoom technology, participants intended to connect with their deeper self, being, or essence, by bringing their I-ego-awareness from the thinking mind, often associated with the frontal part of the brain, to 19 energetic stations in the body including the heart centre. Considering the results from the HeartMath Coherence Model viewpoint, it seems this intended shift leads to cardiovascular phase synchronicity and interconnection of various bodily subsystems, which is also comprehensible based on our bodily development during embryogenesis. Qualitative statements involving feeling also point to increases in well-being, indicating changes in levels and individual transformative experiences as predicted by the Theory of the Six Main Levels of Consciousness. Increased focus, stilling of the mind, and calming of emotions also seem to be health benefit by-products of Intuitive Meditation (IM), but further research on more advanced practitioners is needed. This preliminary study needs to be repeated using a bigger sample size and further research on more advanced IM practitioners is required.

## 1. Introduction

The purpose of the study is to further our understanding of human consciousness through the study of people exploring the nature of their own consciousness using a method of meditation known as Arka Dhyana or Intuitive Meditation (IM). This method involves rewinding our surface consciousness to reverse everything that has happened to us “to experience the very essence of our being, the Self” (Arka, 2006, p. 180). Based on his own exploration into the nature of his own consciousness and that of his pupils using the Intuitive Meditation) method he developed, the yogi and philosopher Arka proposes people will experience six main levels of consciousness when undertaking this inner journey, which has given rise to the Theory of the Six Main Levels of Consciousness [1]. The main levels he [1] identifies are: (1) M (Mind)–Consciousness, (2) SM (Subliminal-Mind)–Consciousness, (3) F (Feeling-Mind)–Consciousness, (4) H (Emotional-Heart)–Consciousness, (5) HS (Heart-Soul)–Consciousness and (6) PS (Pure-Self)–Consciousness. (pp. 37–38). Arka [1] further maintains that practitioners will have individually unique experiences within each level.

When starting the exploration into the nature of one’s consciousness, the person starts where they are now and that usually involves the area of the brain involved with behavior, speech, and reasoning associated with the prefrontal cortex. This area reaches full maturity late [2] and from the evolutionary perspective of Fuster [3] involves the adaptation of the organism to its environment and its relation to other cortices. In his theory, Arka [1] associates M (Mind)–Consciousness, with the “surface of the cerebral region (which) becomes sharpened by the cultivation of learning, evolves into a faculty called intellect” (p. 37). This suggests an overlap between the PFC and Mind-Consciousness of Arka, involving our later development as intellectual beings.

Previous research into the IM method, also known as Arka Dhyana, undertaken by Tina Lindhard for her doctoral thesis [4] and later described in two articles [5,6], showed that participants who attended a five session introductory IM course began a transformational inner journey which was marked by a highly significant shift (at the *p* = 0.001 level) towards a more feeling based consciousness as measured by the Feeling consciousness scale (FCS) she developed for the study. “This scale includes items such as unity, peace, intuition, positivity, awareness of emotions, and connection to one’s inner Self, sometimes expressed as soul, inner being, or atman” [4] (p. 184).

Inspired by the positive findings of Lindhard [4] and Louchakova-Schwartz’ [7] comment that meditation methods that investigate the ‘self’ produce positive characterological transformation which in turn promotes healing including “traces of prior trauma and integrate the shadow” [5] (abstract), the authors of this study decided to collaborate to find out more about the initial shift in level of consciousness mentioned in Arka’s theory by using HeartMath (HM) Inner Balance or emWave2 electronic technology to measure mean coherence and achievement of 6 of the participants before and after each session.

The rationale behind the use of the HM electronic devices may be understood in terms of the HM coherence model that rhythmic activity in living systems reflects information transmitted by interconnected biological, social, and environmental networks [8]. The term coherence implies logical argumentation, harmony, interconnectedness, and consistency, where the whole is always more than the sum of the parts. For example, in IM it can be seen as implying a relationship between various bodily subsystems and related energies and the quality of yoking with divine energy, implicit in the term “yoga” [9,10].

Both IM and HM emphasize the “feeling heart” and increases in intuition, which literally refers to inner understanding and knowing. HM studies distinguish three dimensions or types of intuition; the implicit process of insight, energetic sensitivity in detecting electromagnetic and other environmental signals, and non-local intuition, which transcends conventional space-time [11]. All types typically operate inclusively in that gestalt apprehension experienced as intuition by many people, especially traditional diviners, healers, psychologists, and some medical doctors. Extensive HM studies endorse empirical research with South African traditional healers as to the crucial function of the coherent heart before the brain in intuition. Further archetypal, cultural endorsement of this empirical research is apparent in the similarity of the experiential terms *umbilini* and kundalini, respectively used to describe intuition by Zulu indigenous and yogic practitioners [11,12].

In this study, we were mainly concerned with the initial shift in experiencing consciousness between the thinking mind, which is associated with the surface of the cerebral region and the feeling mind, associated with the heart center, and possibly the experience of other deeper levels also connected with the heart. The heart center is in the middle of the upper chest and is also associated with the spiritual heart that is considered the “psychospiritual centre of embodied consciousness in the interior space of the chest” in [13] (p. 43), but here we refer to the heart in a more open non-specific way. For people already experiencing a certain degree of heart consciousness, the practice of the IM method would enable them to access deeper layers of feeling as outlined in Arka’s theory.

Evaluation of IM method was both process and outcome orientated where practitioners were instructed to rest and record coherence and achievement quantitative data for exactly five minutes before and after each meditation session. These quantitative data were supplemented by qualitative information obtained from participants’ comments concerning (1) how they felt Arka Dhyana had complemented their wellbeing, (2) a general statement outlining their individual experiences while practising the method Arka Dhyana (IM) and (3) a more detailed record of the experiences of the participants after each IM session. The results were analysed and then looked at from the perspective of the HeartMath Coherence Model and the Theory of the Six Main Levels of Consciousness

According to Arka [1], consciousness is the property of the entity or being, whereas awareness is “the effect of consciousness … you can cultivate awareness over a period of time by willfully allowing it to flow or work in a certain direction and can thus increase your knowledge on a subject” [1] (p. 39). Conscious awareness, on the other hand, “takes one to a deeper level. It is not just a matter of raising awareness intellectually through knowledge. Here you raise your awareness emotionally with full involvement of your deeper mind prevailing in the heart region … some people may be conscious but are not effectively aware of their particular presence” [1] (p. 39).

### Hypotheses

Although the IM method intends a shift in consciousness from the rational mind to the emotional heart-mind to the discovery of the self [14], owing to empirical, experiential, and cultural evidence for the role of the coherent heart in intuition, it was also expected that IM participants would experience increases in mean coherence and coherence achievement as measured by HM technology before and after IM practice.

It was further expected that the participants’ qualitative statements of how the IM method had added to their wellbeing, and descriptions of their individual experiences during this experienced shift in their level of consciousness, would also provide interesting information regarding what coherence involves when using the IM method.

The null hypothesis was that IM would not increase mean coherence and coherence achievement as measured by HM technology before and after IM practice.

## 2. Materials and Methods

### 2.1. The Participants

Participants were recruited through a flyer informing people about an Intuitive Meditation course that would take place internationally via zoom technology. This flyer was shared by one of the researchers with the staff of the psychology department of his university and his acquaintances. People who showed an interest were admitted to the course, and those that wished were invited to also take part in a study of the IM method. Out of these, participants who possessed or had access to HM Inner Balance or emWave2 equipment were asked if they would also participate in the study we report in this paper. As the experiment took place during COVID restrictions, we were obliged to do this part of the study this way, hence the sample was by convenience. As the measurement was both process and outcome orientated, the scores of each participant reflected each participant’s change in coherence prior to and after each IM session. This research design excludes the need for a control group involving matching samples.

Sample mean age was 53 years, standard deviation of 16, and age range from 31 to 74 years. Three held doctorates, one masters, and two bachelor’s degrees. There were four professional psychologists, one yoga teacher, and one artist. Five professed to various forms of Christian religion and the sixth to various mystical traditions. For confidential reasons, the six participants were simply coded A to F.

### 2.2. Intuitive Meditation Course Process

Following the establishment of relationship, rapport and appropriate intuition or ambience within the group, the Arka Dhyana Intuitive Meditation process was introduced and progressively, the three pillars of the Arka Dhyana method consisting of touch, breath, and sound, were demonstrated in sequential stages. This demonstration was preceded by practicing the gesture of leading the thinking mind to the area of the heart in the center of the upper chest. This gesture typically involved moving one’s hand slowly from the region of one’s head (thinking mind) to that of one’s heart while maintaining the fingers in *gyan* mudra (i.e., the thumb and the index figure forms a circle) and inviting the thinking mind to come on a journey. The course as presented consisted of practising touching rhythmically 4, then 8, 12 and 19 body parts, consisting of feet, calves, knees, thighs, sacrum, naval, solar plexus, heart, shoulders, elbows, hands together, throat, mouth, nose, cheeks, ears, forehead, and head, where each was accompanied by a syllable of the mystical vibratory sound, SAROOGOVAUM. Except for the first session consisting of 3 h, each of the following sessions lasted 2 h. Each stage is built sequentially on the previous stage. Finally, in the fifth session, physical touch was superseded by visualized touch with hands of light.

In IM, the modulation of the vibratory sound SAROOGOVAUM is directed at each energetic station, and participants are instructed to increase its pitch and duration as they ascend the body synchronizing each movement with the breath. By lengthening the sound, the out-breath is encouraged to become more and more prolonged. This practice sometimes initially facilitates the shadow emotions to surface, during which the practitioner is invited to become one with each station touched, as well as the arising emotion that is seen as a part of emotional consciousness but is not the whole of the self.

The IM introductory course lasted 11 h but, between sessions, participants were invited to become more familiar and involved with the IM method by practising it.

### 2.3. Intention

The main intention in IM is to discover our true nature or deeper self. For this reason, participants are invited to launch a desire to connect to it when practising IM. It is also pointed out that IM is like a musical instrument and can be played however the participant wants. “The preliminary task of setting one’s intention is the thrust of the IM method” but people may utilize it how they like. Some people want to “improve their health or feel good, whereas others want to discover and connect with their true nature or inner Self” (S. Arka, public unpublished talk, Ecocentro, Madrid, Spain, 6 August 2016, in [3]) (p. 15). The latter was the suggested intention in this study.

The IM intention can be contrasted with that of HM, which typically focuses on coherence, through intentionally slowing heart and breath patterns, while cultivating positive, renewing feelings such as peace and love.

### 2.4. Instrument

HM emWave2 and Inner Balance electronic devices measure and monitor psychophysiological coherence, which emerges from the harmonious activity and interactions of the body’s subsystems. Physiological coherence is associated with a smooth, sine wave-like pattern in heart rhythms and a narrow-band, high-amplitude peak between 0.04 and 0.26 hertz in the low frequency range of the heart rate variability (HRV) power spectrum. This is associated with general well-being and optimal performance [11]. Coherence means and achievement totals were used in this study. The Inner Balance and emWave2 electronic devices are unique in their potential to measure insight, energetic sensitivity, and non-local intuition. HM and related studies repeatedly affirm the influence of the coherent heart on the human brain, social relationships, and wider ecology [11].

Initially, from a purely psychophysiological perspective, coherence reflects cardiovascular phase synchrony, which includes the order in the relationship between heart and breath rhythm patterns, as well as other oscillating physiological systems, such as blood pressure, brain waves, cerebrospinal fluid, and emotion, all of which are influenced by the heart, which is the greatest physiological oscillator, the conductor of the orchestra so to speak. Correlated variables include the heart’s electromagnetic field, oxytocin; the love hormone, and neurochemicals, such as serotonin and dopamine. HM studies have shown that higher coherence results from positive renewing emotions independently of cardiovascular rhythm; in real (lived) life, all are intimately interlinked. An important naturally occurring variable is respiratory sinus arrhythmia (RSA), whereby inhalation and exhalation are associated with increasing and decreasing pulsation, respectively.

The electronic Inner Balance app contains a photoplethysmogram (PPG), which uses a light-emitting device (LED) to monitor blood volume changes from light absorption. Photoplethysmography sensors are usually attached to the earlobe. The changes are monitored on a downloaded app in a smartphone, such as an iPhone or any Android device [8,15].

### 2.5. Data Analysis

The small convenience sample recorded HM Inner Balance Coherence Mean and Achievement records of 39 pre-test and post-test IM practice sessions. This indicted a simple Wilcoxon Z nonparametric statistical analysis of related samples of data. Unfortunately for technical connectivity reasons, participants A and B could not participate in all five IM sessions, so reported only limited quantitative and qualitative data, as will be apparent in the results that follow. The small sample ensured that all participants’ experiential descriptions could be included in the thematic content analysis and related discussion.

### 2.6. Ethical Statement

The study was approved by the Academic Committee of the International University of Professional Studies (Project Lindhard, 16 March 2021); all appropriate ethical standards as required by the Helsinki Declaration and Professional Boards for Psychologists applied. The consent form preceded the submission of the online answers to the open questions and had to be agreed on before proceeding with the questions. Confidentiality of the participant’s data were sustained during the study by numbering them alphabetically. Participants were regularly informed that if they needed any help with the arising emotions or clarification concerning the practice or their experiences of Arka Dhyana (Intuitive Meditation)—or if they would like to share anything about how their life was unfolding, they were more than welcome to contact Dr Lindhard via e-mail or WhatsApp. Regular contact between individual and group participants and Dr Lindhard was maintained throughout the course.

## 3. Results

### 3.1. Quantitative Findings

Because they occurred before and after any IM session, quantitative findings essentially refer to ongoing process evaluations of the IM course. These consisted of coherence and achievement mean scores and related statistical findings, as appear in Table 1.

Table 1 refers to 39 comparisons of coherence and achievement mean scores (with standard deviations in parentheses), of HM Inner Balance recordings as taken by 6 participants before and after 39 Intuitive meditation sessions. Non-parametric comparisons of these 39 related data samples using Wilcoxon Z statistics indicated highly significant increases in both coherence and achievement associated with these Intuitive Meditation sessions (*p* < 0.0001).

### 3.2. Qualitative Findings

Qualitative findings rest on answers to the two reflective questions which were collected online as part of the Revised Feeling Consciousness Scale as well as a record made by participants of their experiences after each IM session and shared with the researchers at the end of the course.

### 3.3. Qualitative Answers

As this study only involved a few participants and their answers were concise, answers to question 1 are given in full. (Participant codes appear in parentheses).

Question 1: “In what way or how do you feel Arka Dhyana has complemented to your wellbeing?” The IM method was said by participants to increase wellbeing as it led to:

“a recognition and acceptance of different parts within me and that is ok” (D)

“helped to still my mind and calm and still the agitation and anxiety in my body, bringing me into the body and heart-focus more easily” (F)

“brought me back to a place of remembrance of how much more I am than this body and this experience on earth. Through my daily challenges I now have something to continuously return too, that helps me feel less stressed and more connected to myself and my higher power resulting in me flowing through challenges with more grace and acceptance” (C)

“more aware of many aspects of light love and life. It is easily amalgamated into my existing meditative and contemplative practices” (E)

“opened a new way of approaching my inner being that I feel I need to connect with more so it’s a positive move in my life” (A)

One subject also reported “not being sure as yet” of how the method had benefited her (B)

Question 2: “Please add something about your experiences while practising the method Arka Dhyana (IM), and how you feel afterwards.” As expected, the experiences reported during this initial shift in consciousness from the rational mind to the feeling heart-mind were highly individual. Four of the six participants gave concise one or two-sentence type answers, whereas the 5th and 6th included the record of experiences noted after all 5 sessions.

Reported personal experiences include:

“I have become aware of energies and vibrations externally of me and in other people. It is made me MORE sensitive to that energy I feel in other people” (D)

“I noticed that being in the group practice space would bring up a kind of jittery feeling and agitation that would settle by the end of the practice. I was able to observe how busy and agitated my mind is. I was surprised at how it works so well; I didn’t expect that. The sounds and sensation helped to focus my mind so that it didn’t drift off and dissociate as is its usual habit. I am still impatient with the whole procedure when I do it by myself. The final session helped me to experience the process more viscerally, especially experiencing the spirit body so clearly. I practiced that this morning. It does help me to feel centred and integrated and aligned in my heart, soul and body after practising” (F)

“It helped me focus (B)

“I generally feel very relaxed after practising and hungry for some unknown reason. But I for sure feel more connected to my body and my soul. I have a groundedness and surety of myself and my decisions (C)

I felt an escape from everyday problems (except when the unstable internet cut us off in sessions). It was a relief to remind myself there is a way to find this peace and time for connection. I still have difficulty quietening my thoughts but have this as a much-desired goal” (A)

Record of experiences (3). Although the participants were asked to record their individual experiences after each IM session, three participants (A, B and F) gave limited, one sentence or phrase type feedback on their personal experiences. One participant (C) reported fully on all five sessions, providing a valuable illustrative qualitative example of transformative personal change. This participant C’s experiential descriptions associated with each of the five Intuitive Meditation instruction sessions (with session numbers indicated in parentheses) follow verbatim:

Participant C (1) A sensation of a loving, gentle touch that I crave from another, I find I can give it to myself through the method of meditation. Connecting to my body and creating a relationship with parts of my body that I haven’t had before- it feels new and comforting. (2) This takes time and showing you daily to my practice like all relationships. Body and soul are all one. A lot of tingling through my body almost scary- mostly my feet. It got extremely strong and then I broke through to a place of peace, surrender and groundedness. (3) Feeling immense gratitude for this session- it felt like a breakthrough into the present moment where u was feeling really agitated before the session and I didn’t know where to put my mind. (4) I was feeling really tired before starting the session, found the session challenging to keep going but I relaxed my body—jaw, stomach and shoulders coming back to my breath. A lot of electric feeling on the right side of my brain. Feeling really present and in the moment. An acceptance of where I am in the moment, which is a sadness. Less resistance and more connected to my body and essence. (5) Finding more acceptance of my true self that includes the not so pretty parts of selfishness and self-centeredness. They all belong and need to be seen to lose the power. Less judgement and more compassion for myself. Less whipping myself and more allowing things to be without judging them as good or bad.

Two participants (D and E) gave very full feedback in the form of artistic and phenomenological reports, respectively, that are beyond the scope of the present study. A summary of D and E’s descriptions follows:

Participant D. “I have become aware of energies and vibrations externally of me and in other people. It is made me MORE sensitive to that energy I feel in other people and in myself.

The experience was overwhelming, and I found myself close to tears. I have not managed to ‘feel’ emotions for so long and suddenly I became aware of turmoil … it is exactly how it felt on day 1. Big mess of swirling colours and lines and blurs.

I am a very visual person and not good with words, or parts of me struggle to verbalise what I want to say. I think it is a way of “blocking” that which I do not want to confront. I like the idea of parts of me, needing to be free, attended to, waiting to be heard.

I, my ‘messiness’ coming together. I could visualise the end product of a creation.

Two parts of the pot, Yin and Yang on opposite ends of the base, black and white circles built out of stones or pebbles, big stones and smaller stones, can both mean before and after. Smaller stones in the ‘white’ part represent the little things that come into our lives/hearts, good or bad. The black part consists of mainly the big things in my life, good or bad.

While ‘moving between the parts’, healing between the parts and connecting between the parts, bending and shaping the tree (using IM methods throughout the various sessions).

I still feel a deeper connection to my bonsai garden, where I am happiest. I am more aware of the insects visiting, the snakes and little birds that come and play. I find myself humming SaaRooGoVaum as I like to feel the vibrations in my chest near my heart. Every day is a learning curve for me as more and more opens up for me”.

Participant E. Personal experiences of this participant were recorded after each IM session and summarized as follows: “There are different layers and levels of heart consciousness involved in intuition; personal, social, and global, life, love, and light. Hands of light made me more aware of mainly, old, sport injury issues on the right side of my body, which I am healing further: hip, lung, shoulder, throat and eye are all getting extra love and care. Socially our group gelled more in the fourth session. Many processes seemed similar to other group processes: intention, expectancies, attitudes, motivations, relationships, energy, sentience, awareness, consciousness. I have included other variables in my earlier, experiential descriptions. At the lower collective consciousness level, dreams, which initially flooded through, were represented by more than usual reptilian imagery, e.g., crocodile and snake, spiritual sexual imagery, which were energetically transmuted into intuition, love, empathy, and compassion. Inner *umbilini* kundalini awakening, *isangoma* animus, embryology, Jung’s [16] active imagination, Sufi imaginal world, phallic clitoral physicality, lower-level animal unconsciousness, ancestral calling, feet-sand running, integration of sea and river ancestors, rewinding for fast forward planet generational healing. Traditional spiritual images included Zulu *isangoma*, the black Madonna of Monserrat and related African and Spanish geographical and historical associations, typically divine and feminine, as related to our group who were predominantly women. I found IM similar to my usual meditation and comfortably used personal, Christian, ancestral, HM and IM mantra interchangeably during practice. Differences noted was that my usual meditation, contemplation and/or prayer is relatively less past, whole body and Self-orientated and more psychophysiological, heart, personal, group, global and future-directed. Both IM and MH are concerned with whole making, IM is essentially more orientated towards knowing and HM towards healing. However, these orientations appear inextricably interrelated”.

## 4. Discussion

So, what do the significant coherence findings mean and involve, especially as the intention of the IM method is not coherence as in HM but connection to our self, or inner being? In addition, the HM and IM methods are uniquely different. By prolonging the breath, IM initially sometimes facilitates the shadow emotions to surface, during which the practitioner is invited to become one with each station touched as well as the arising emotion that is seen as a part of emotional consciousness but not his or her identity, and on the other hand, during the HM method the breath pattern is deepened, and positive feelings such as love and peace are cultivated. From a similarities perspective, both IM and HM are concerned with feeling, HM through specific heart centeredness, while breathing and noting, experiencing or generating feelings, while, in IM the practitioner, based on direct experience, becomes consciously aware of what is happening in their inner world, including arising feelings, emotions and sensations.

The findings can be viewed as applicable at various levels of sentience, awareness, and consciousness from an HM perspective. From an embryo model perspective, the Inner Balance application (app) works directly with the heart system, both autonomic parasympathetic and sympathetic divisions, as well as the central nervous system (CNS), especially the vagus nerve with its 80% afferent fibres going from heart to brain via the amygdala and prefrontal cortex. Physiologically, cardiovascular synchronization entrains all other physiological oscillatory systems as well as other organ systems, immunological, hormonal, biophysical, neurochemical, and electromagnetic systems. At the psychological level, coherence is related to positive and renewing emotions, ranging from appreciation to serenity and love to happiness and ecstasy. Interpersonally, coherence implies alignment and synchronization in interpersonal and group processes. For example, the present study was associated with the development of typical group processes, such as openness, trust, and freedom to express emotion verbally and in writing. Although the focus was on embodiment and embodied spirituality, broader, wider spiritual apprehensions also applied. 

From an IM understanding, the behavioural gesture of taking our thinking mind to the heart area coupled with the desire to connect with the inner being or soul at the beginning of the IM practice opens us to a change in the level of consciousness from the thinking mind–consciousness associated with the frontal part of the cortex to feeling mind–consciousness associated with the heart. Looking at the development of the body during embryogenesis, and summarizing from Lindhard [2], the heart system develops first with blood appearing in the ectocyst, which then runs in capillaries to the cranial end of the germinal disc and then back to the ectocyst via other capillaries. The central point where the blood starts its reverse journey, “takes on a rhythmical character (which) is the first indication of the origin of the heart” [17]. Hence, from a physiological point of view, the heart is the primary system. However, through pulsation which starts at the cranial end of the germinal disc, the incarnating entity is linked to the underlying core principle and property of universal existence, cosmic existence, and local existence [Arka in Lindhard 2] and therefore to the Absolute which in its’ creating form, is perpetual spanda [18] (p. 10) or creative pulsation. This is consistent with Planck [19] who declared:

There is no matter as such. All matter originates and exists only by virtue of a force that brings the particle of an atom to vibration and holds this most minute solar system of the atom together … We must assume behind this force the existence of a conscious and intelligent mind.

Connecting to one’s inner being via our primary or Mother Mind linked with the heart center, not only connects us to the Absolute, but to all creation which is pulsating. One participant (E) playfully and alliteratively described this coherence interconnectedness and/or oneness as an embodied, whole making, symphonic synchrony, of spirit, soul, self, sky, sea and silence.

On another level, summarizing Lindhard [20], intraembryonic mesoderm gives rise to the major structural components and organs of the inner body, including the notochord (made of three-dimensional meso tissue) which according to most researchers, pattern the central nervous system (CNS) from below. This adds emphasis to the idea that the mesoderm is primary, and the neural system is secondary. At best, de Bree, de Bakker, and Oostra [21] suggest they might be interrelated, which leads to the insight that the body is a dynamic, interconnected (or entangled) whole.

Although the mesoderm structures are to do with function and are operational at birth, whereas the prefrontal cortex (PFC), develops late, both phylogenetically and ontologically [22], and even though this region shows developmental changes in the first year of life, it only reaches full maturity in humans {2]. However, when we develop our intellectual abilities associated with this area of the brain associated with thinking Mind–Consciousness, we may lose contact with the inner mesoderm layer which according to van der Wal [17] is incorrectly named as it is not a limiting skin but simultaneously “creates space and connects” (p. 42). Hence, by bringing our awareness from the neural part of the brain with its emphasis of thinking about the world, to the heart area and different energetic stations in the body, we regain our connection to the deeper inner layer, which is unfolding and functioning in the now, somewhat reminiscent of Tolle’s [23] emphasis on the Power of the Now A Guide to Spiritual Enlightenment. 

In IM and HM meditation, practitioners tend to withdraw their secondary perception system that involves the five basic senses through which information is gained about objects in the outside environment and open their body-based awareness, which, along with the other vestigial senses, is primary [20]. It is suggested here that by regaining connection to the pulsating heart, this primary subsystem, consisting of inner three-dimensional tissue unfolding in time, helps raise our conscious awareness and deeper mind prevailing in the heart region. At the same time, this connection probably helps bring the whole psychophysiological system into coherence.

Touch is often referred to as the mother sense as it is primary and although the importance of touch as an energy exchange between people has been established [22], the effect of touching oneself, as in the Arka Dhyana method still needs to be researched. However, participant C’s statement regarding self-touch as “a sensation of a loving gentle touch that I crave from another I find I can give it to myself through the method of meditation. Connecting to my body and creating a relationship with parts of my body that I haven’t had before- it feels new and comforting”. This qualitative statement indicates self-touch as practiced during IM might be a valuable ingredient in bringing harmony between subsystems, but that needs further research.

Participants’ experiential responses varied appreciably in depth and detail. A shift in consciousness from our rational thinking mind to a more feeling based consciousness associated with the heart and body is reflected in the comments: “I for sure feel more connected to my body and my soul, … Connecting to my body and creating a relationship with parts of my body that I haven’t had before- it feels new and comforting … less resistance and more connected to my body and essence (C); The practice helped to still my mind and calm and still the agitation and anxiety in my body, bringing me into the body and heart-focus more easily” … The final session helped me to experience the process more viscerally, especially experiencing the spirit body so clearly (F); Hands of light made me more aware of mainly, old, sport injury issues on the right side of my body, which I am healing further old, sport injury issues on the right side of my body (E). 

Although the other participants did not specifically mention the body or feeling, participant B’s single comment that the method helped her focus is highly relevant as increased focus is a very important aspect of the IM method as it leads to the dhyana experience. Focus was also mentioned by another participant (F) when reporting “the sounds and sensation helped to focus my mind so that it didn’t drift off and dissociate as is its usual habit”. 

Some participants went further and awakened level 4 Emotional-Heart –Consciousness mentioned in Arka’s theory. This was indicated by the acceptance of shadow emotions as different parts leading to “calm and stilling of anxiety (and other emotions) in the body”. The acceptance of these emotions, often classified as negative in a non-judgmental way, awakes participants to other arising emotions like gratitude. Participant’s descriptions of the IM course resonated with appreciation, a positive, renewing and paradoxically, often unappreciated emotion, which has been given due recognition in HM studies [11] and has interesting methodological and contemporary connotations [24,25].

Furthermore, spiritual levels were touched on by some participants reflected by participant D’s answer, which can be summarized by a shift to a full-blown emotional level of consciousness, which allowed her to tap into the deeper energetic level and increased creativity. Other changes to a deeper and more spiritual level are recorded in the statements as having a “remembrance of being more than the body, more connected to the self and higher power, recognition of many aspects of light, love and life and the opening of a new way of approaching ones’ inner being, kundalini awakening and many intuitive personal insights. 

Participants’ (D and E) artistic and phenomenological reports provide enough depth and detail for two separate studies. Depth psychologists emphasize the immediate total sense of the whole that characterizes the intuitive experience [16,26]. From an experiential perspective, Assagioli [26] notes that intuitions enter consciousness via levels of personal and/or collective unconscious in two ways: The first involves a conscious welcoming openness; the second is more akin to a revelatory flash of lightning. The experiences of participants D, E, and F emphasize both intra-psychological (between the conscious and the unconscious) and transpersonal features (from the collective unconscious). Similar findings are reported in other studies [27,28].

Along with these common themes coherent with a body-based feeling consciousness, participants emphasized the pathic nature of their experience in terms of feelings, energy, sentience, awareness, consciousness, the self, body, intuition, life, soul, spirit, the group, interconnectedness, love, knowing, wholeness and healing, participants expressed this commonality through their unique highly differentiated participant responses. From a broader conceptual perspective: (a) It seems yogic in emphasis on breath and chakra like focus on 19 body parts, some related to chakras *manipura*, *anahata*, *vishuda* and *ajna* [7,8,29], (b) Its bottom up, feet to head, kundalini like sequence resembles African *isanusi* divination and healing *umbilini* breathing meditation, as described by Mutwa [30], (c) The hands of light sequence resonates with chi kung stillness in movement and microcirculation of light [31], Johrei energy healing sequences [32], Brennan’s [33] High Sense Perception and healing hands of light, as well as aspects of many other heart-based meditation techniques summarized by such writers as Benson [34,35], Louchakova [36] and Wilber [37,38]. 

### Limitation of the Study

The varying familiarity with HM method might be a confounding variable but as the Independent Variable for this study was the practice of the IM method this should not be a bias especially as the assessors were instructed to simply use their electronic devices as neutral assessment tools for exactly five minutes before and after each session. The qualitative comments indicate that the participants were practicing IM and letting the different emotions and feelings emerge such as the ‘overwhelming’ emotional experience of participant D, the stilling of anxiety of F as well as the sense of connection to their inner being or self of participant A and C. Participant’s B’s unsurely of the effects of the methods is also an indication of somebody starting a journey where admitted increased ‘focus’ is an important aspect when one shifts one’s consciousness from outside to inside. That participant E felt that this practice was compatible with the HM practice, does not mean that the effects of IM were not being measured during the research, as participants were specifically told not to practice during measurement sessions. This participant’s qualitative statements also indicate that IM moved different energies and visions to those the assessor was accustomed to.

Another confounding variable is the sample was small and of convenience but as it is a new area or research, a small sample has its advantages in seeing if the topic is worthwhile pursuing. Studying the nature of one’s own consciousness is a lifelong journey and normally reported from the first-person point of view through phenomenology. In this study, we, as researchers, are studying people studying the nature of their own consciousness using the IM method. Hence, gathering the data from 6 people instead of one person reporting on the changes of their own consciousness is a step forward. That the sample consisted of professional psychologists is not an advantage. The fact that they know a lot about what is said about consciousness often makes it more difficult when showing them how to explore their consciousness. Knowing a lot about a strawberry from a scientific point of view is not the same as putting it in your mouth and tasting it, which is purely experimental; the same applies when exploring our own consciousness when learning to go below our thinking mind.

Another shortcoming is that two participants did not complete the course and did not complete the post-test. One dropped out after the first session saying he found the method difficult and not what he expected and the other was moving house and did not complete the course. The small sample makes it difficult to generalise to a wider audience.

## 5. Conclusions

Although this research has admitted shortcomings, as a preliminary study it points to the connection between coherence and the reported feelings of wellbeing expressed by the participants on learning the IM method. In addition, as the reported qualitative statements regarding how IM has added to their wellbeing suggest a shift in levels of consciousness as described in Arka’s theory of the Six Main Levels of Consciousness, it poses the interesting question of whether the development of our intellectual mind disconnects us from our inner body and our heart-based intuitive nature. Although outwardly very different in philosophy, it seems HM coherence is a concept that adds additional insights to what is happening during the IM experience, and possibly, the Theory of the Six Main levels of Consciousness might provide further insights concerning the HM model as both are heart-based methods. This venture of looking at the data using the HeartMath coherence model, and Arka’s Theory of The Six Main Levels of Consciousness adds new insights to both perspectives on a theoretical and practical level, as well as aiding our knowledge of human consciousness, different levels of mind and coherence.

To summarize, this limited phenomenological and experimental evaluative study attempts to understand more about human consciousness based on direct spiritual experiences when one starts the inner journey into its exploration using the IM method in which HM technology not only seems an exciting way to help validate reported changes in consciousness that are described by participants as leading to increased wellbeing related to feelings, stilling of the mind and calming emotions but shows meaningful physiological changes take place involving harmonious activity, interconnection of the body’s subsystems and cardiovascular phase synchrony. Although IM is very different in method to the focused attention meditation training described by Lenhart, Steiger, Weibel et al. [39] future research into cortical reorganization of participants after learning the IM method could help point to the link between the heart, coherence, levels of mind, wellbeing, and different neural organization processes.

This study needs to be repeated using a bigger sample size and further comprehensive research is needed to understand more about the IM method and the levels a practitioner undergoes or his or her inner journey from the rational mind to the emotional heart-mind to pure consciousness which is said to result in ultimate wellbeing or enlightenment.

## Figures and Tables

**Table 1:** Quantitative Process Evaluation of the IM course.

Assessment	Number	Coherence	Achievement	Wilcoxon Z	Significance
Pre-test	39	1.79 (1.54)	99.77 (91.20)	4.249	0.000
Post-test	39	2.84 (1.56)	190.18 (96.87)	4.354	0.000

## Data Availability

Supporting data details are attached: Arka Dhyana (IM)—HeartMath Coherence and Achievement, Pretest and Postest Data.xlsx; Meditation—HeartMath study Participant Codes and Qualitative Dta.xlsx.
